# Current and emerging artificial intelligence applications for pediatric musculoskeletal radiology

**DOI:** 10.1007/s00247-021-05130-8

**Published:** 2021-07-16

**Authors:** Amaka C. Offiah

**Affiliations:** 1 Department of Oncology and Metabolism, University of Sheffield, Damer Street Building, Sheffield, S10 2TH UK; 2 Department of Radiology, Sheffield Children’s NHS Foundation Trust, Sheffield, UK

**Keywords:** Artificial intelligence, Bone, Children, Musculoskeletal, Pediatric radiology

## Abstract

Artificial intelligence (AI) is playing an ever-increasing role in radiology (more so in the adult world than in pediatrics), to the extent that there are unfounded fears it will completely take over the role of the radiologist. In relation to musculoskeletal applications of AI in pediatric radiology, we are far from the time when AI will replace radiologists; even for the commonest application (bone age assessment), AI is more often employed in an AI-assist mode rather than an AI-replace or AI-extend mode. AI for bone age assessment has been in clinical use for more than a decade and is the area in which most research has been conducted. Most other potential indications in children (such as appendicular and vertebral fracture detection) remain largely in the research domain. This article reviews the areas in which AI is most prominent in relation to the pediatric musculoskeletal system, briefly summarizing the current literature and highlighting areas for future research. Pediatric radiologists are encouraged to participate as members of the research teams conducting pediatric radiology artificial intelligence research.

## Introduction

Simply put, artificial intelligence (AI) can be defined as software that automates (or semi-automates) a cognitive task. AI applications in the musculoskeletal system can be fully automated (e.g., bone age assessment) or semi-automated (e.g., vertebral fracture assessment). Although AI tools in general can be categorized as AI-assist (helping the radiologist), AI-replace (replacing the radiologist) or AI-extend (exceeding the capability of the radiologist) [1], as far as the author is aware, no clinical tool functions in the AI-extend mode in pediatric musculoskeletal radiology practice.

Existing AI tools can help to improve image quality; aid in the measurement of lengths, angles and volumes; or aid in the detection of pathological processes, the last through recognition and classification of morphological or textural abnormalities.

Of the 144 AI products that are CE (Conformité Européenne) marked and commercially available, 74 also have United States Food and Drug Administration (FDA) approval and 18 are related to the musculoskeletal system [2]. Of these 18, one is for image enhancement and post-processing rather than being a diagnostic aid, per se. Considering the remaining 17 AI musculoskeletal products, the majority (14) have been designed for aiding diagnosis from radiographs, while use in pediatric radiology is only explicitly stated in the information available for 3 of the 17 tools. Table 1 summarizes these 17 available musculoskeletal AI tools; all 3 tools intended for use in pediatric radiology are for bone age assessment [2].

**Table 1:** Currently available CE-marked musculoskeletal radiology artificial intelligence (AI) tools^a^

Date on market	Company	Product name	Disease targeted	Modality	Pediatrics^b^	CE class	FDA
Mar 2009	Visiana (Hørsholm, Denmark)	BoneXpert	Bone age	Radiography	Y	I	N
2012	Medimaps (Geneva, Switzerland)	TBS iNsight (Osteo)	Osteoporosis	Radiography	N	IIa	II
Nov 2017	Aidoc (Tel Aviv, Israel)	C-Spine	C-spine fracture	CT	N	I	II
May 2018	VUNO (Seoul, Korea)	VUNO Med-BoneAge	Bone age	Radiography	Y	IIa	N
Jan 2019	QUIBIM (Valencia, Spain)	2D Bone Microarchitecture QTS Score	Osteoporosis Oncology Osteopenia	Radiography	N	IIa	N
Jan 2019	QUIBIM	2D Bone Microarchitecture QTS Score	Osteoporosis Oncology Osteopenia	CT, MRI	N	IIa	N
Jan 2019	QUIBIM	Cartilage T2 Mapping	Osteoarthritis Degeneration Sports diseases	MRI	N	IIa	N
Jan 2019	QUIBIM	Texture Analysis	Tumors Osteoporosis Osteopenia Osteoarthritis	Radiography, CT, MRI, PET, US, SPECT	N	IIa	N
Jun 2019	AZMed (Paris, France)	Rayvolve	Fracture	Radiography	N	I	N
Aug 2019	ImageBiopsy Lab (Vienna, Austria)	IB Lab KOALA	Osteoarthritis (knee)	Radiography	N	I	II
Nov 2019	Radiobiotics (Copenhagen, Denmark)	RBknee	Osteoarthritis (knee)	Radiography	N	I	N
Feb 2020	Arterys (San Francisco, CA)	Chest/MSK AI	Fracture Dislocation	Radiography	N	IIa	N
Mar 2020	Gleamer (Paris, France)	BoneView	Fracture	Radiography	N	IIa	N
Oct 2020	ImageBiopsy Lab	IB Lab HIPPO	Hip measurements	Radiography	N	I	N
Oct 2020	ImageBiopsy Lab	IB Lab LAMA	Leg geometry	Radiography	N	I	N
Nov 2020	ImageBiopsy Lab	IB Lab PANDA	Bone age	Radiography	Y	I	N
U	Zebra (Kibbutz Shefayim, Israel)	Bone Health	Vertebral compression fractures	CT	N	U	II

*CE* Conformité Européene, *FDA* United States Food and Drug Administration approval, *MSK* musculoskeletal, *N* no, *PET* positron emission tomography, *SPECT* single-photon emission computed tomography, *U* unknown, *Y* yes

^a^Derived from [2]

^b^Only includes those tools for which pediatric use is explicitly stated

Although commercially available tools are currently only for bone age assessment, ongoing and published research pertains to tasks such as fracture diagnosis (appendicular and vertebral), scoliosis and leg-length discrepancy measurements. Other areas where pediatric research is being performed include determining bone health using the bone health index and diagnosing metopic craniosynostosis and developmental dysplasia of the hip. These emerging applications could achieve commercial release within the next decade.

This review also identifies and briefly discusses areas in which very little AI research has been conducted but in which there is potential for AI to play a significant role; these areas include inflicted injury (child abuse) and skeletal dysplasias.

The main focus of this article is on diagnosis/detection of pathology. For AI applications related to image-quality improvement, image post-processing, quality control, etc., the reader is directed to other articles in this special issue and to the 2019 review by Koska [3].

## Current applications: bone age assessment

Although three bone age assessment AI tools are on the market, the oldest and probably best known is BoneXpert (Visiana, Hørsholm, Denmark). Indeed, BoneXpert is the oldest musculoskeletal AI-replace software tool on the market (Table 1), and more than 150 departments are using BoneXpert in day-to-day clinical practice across Europe, each performing more than 100 analyses per year (personal communication with H.H. Thodberg and P. Bak, November 2020).

**Fig. 1:**
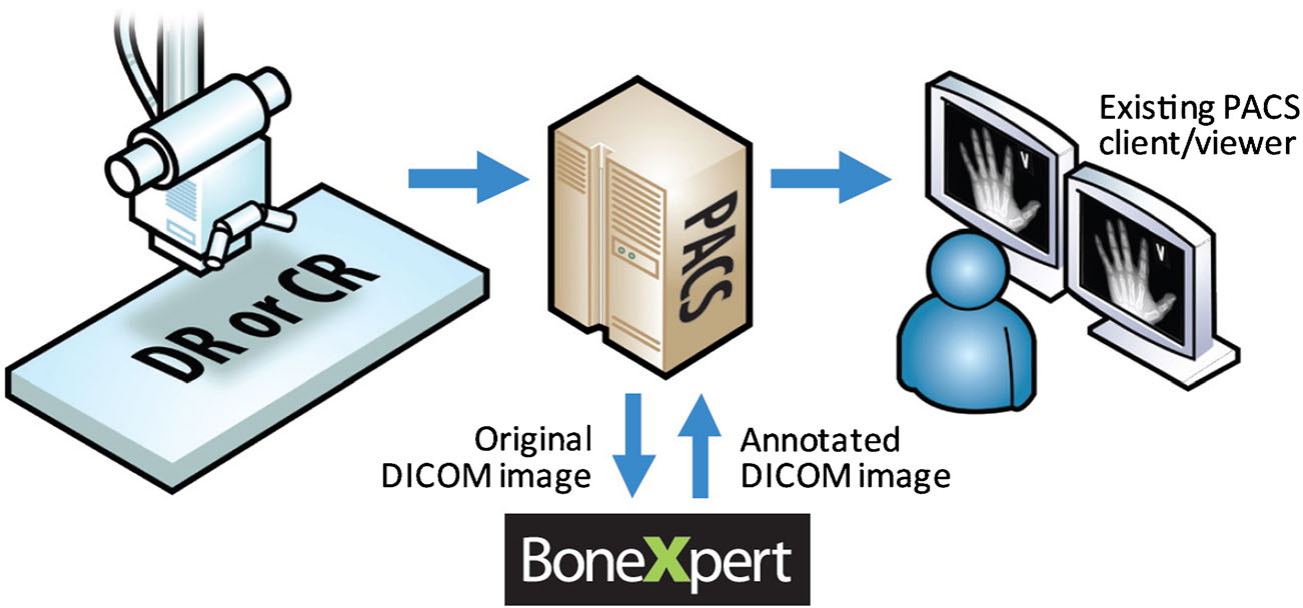
BoneXpert workflow. The radiographer who has performed the left hand and wrist radiograph sends it to the picture archiving and communication system (PACS) in the usual way. The reporting radiologist (or radiographer) transfers the image to BoneXpert, where the automated bone age estimation is performed. BoneXpert then returns the annotated image to the PACS. The reporting radiologist now not only has access to the BoneXpert-derived Greulich and Pyle and the Tanner and Whitehouse 3 bone age assessments, but he or she can also review the original radiograph for disease-related abnormality (e.g., evidence of a skeletal dysplasia). Image courtesy of Peter Bak and Hans Henrik Thodberg (Visiana). *CR* computed radiography, *DICOM* digital imaging and communications in medicine, *DR* digital radiography

**Fig. 2:**
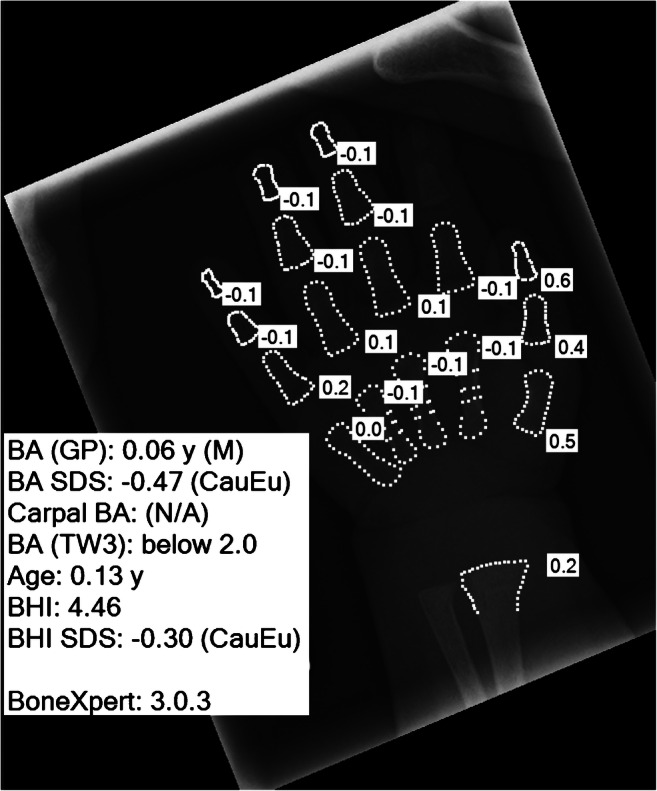
Posteroanterior left hand and wrist radiograph in a 3-week-old boy, following interpretation of bone age by BoneXpert. The figures in the small white boxes represent the Greulich and Pyle bone ages of the individual bones. *BA (GP)* Greulich and Pyle bone age (gender), *BA SDS* Bone age standard deviation score (ethnicity), *BA (TW3)* Tanner and Whitehouse three-bone age, *BHI* bone health index (digital X-ray radiogram), *BHI SDS* bone health index standard deviation score (ethnicity), *CauEu* Caucasian European, *M* male, *N/A* not available (no ossified carpal bones in this child), *y* years. See also Table 2

BoneXpert automatically calculates bone age according to the Greulich and Pyle and the Tanner and Whitehouse standards in a process that takes less than 15 s per hand and wrist radiograph. The method is based on traditional machine-learning methodology and involves prediction of bone age based on shape, intensity and texture scores as derived from principal component analysis. It is worth noting that there are no General Data Protection Regulation (GDPR)-related issues, because BoneXpert is configured as a Digital Imaging and Communications in Medicine (DICOM) node for local picture archiving and communication systems (PACS) and is an image-analysis application only. In other words, BoneXpert does not store data, share data or transfer data outside the local PACS. The pathway and output are illustrated in Figs. 1 and 2, respectively, and the terminology used in the output is explained in Table 2.

**Table 2:** BoneXpert outputs

Term	Interpretation
BA (GP)	Greulich and Pyle bone age (gender)
BA SDS	Bone age standard deviation score (ethnicity)
BA (TW3)	Tanner and Whitehouse three-bone age
Age	Chronological age
BHI	Bone health index (digital X-ray radiogram)
BHI SDS	Bone health index standard deviation score (ethnicity)

Although launched in 2009 as an AI-replace tool, approximately 70% of departments that have the software installed use BoneXpert as an AI-assist tool (A. Offiah, unpublished work). The reason for this is simple: while BoneXpert rejects radiographs with significant abnormality (e.g., poor positioning, abnormal bone morphology, poor image quality), it does not reject radiographs with subtle abnormality of morphology (e.g., early rickets) or abnormality of texture (e.g., metaphyseal striations). If a radiologist does not review the radiographs, then these subtle changes will be missed. The detection of such abnormalities is outside the scope of the software as developed, and radiologists are advised to bear this in mind.

The percentage of radiographs rejected by BoneXpert because of abnormal anatomy depends on the types of patients seen, ranging from approximately 0.4% in general hospitals to up to 3% in hospitals specializing in skeletal dysplasias. The percentage of radiographs rejected by BoneXpert because of poor image quality is generally very low (reflecting radiographer competence). However, departments in which significant edge-enhancement is applied as part of the post-processing of images might see rejection rates of up to 10%, accompanied by the error message, “Too sharp” (personal communication with H.H. Thodberg and P. Bak, April 2021). The software has been tested in multiple populations and ethnicities, including Caucasian, African American, Hispanic, Asian Chinese and Saudi Arabian populations [4–9]; while generally applicable to all ethnicities, some caution is advised, but this is related to the applicability of the standards (Greulich and Pyle, Tanner and Whitehouse 3) and not to the BoneXpert software itself. The latter claim can be made based on diagnostic accuracy studies that have compared manual to automated bone age assessments [10–17], with reported root mean square errors being as low as 0.63 [18].

In 2017, the Radiological Society of North America launched a Machine Learning Challenge, making freely available a set of more than 14,000 hand and wrist radiographs [19]. The best-performing entry achieved a concordance correlation coefficient of 0.99 and differed from ground truth by only 4.3 months, compared to 7.3 months for radiologists [20]. It is possible for readers to test the application for themselves but note that it is for demonstration purposes only (i.e., it is not for clinical use) [21].

Other bone age tools have also been tested and found to be reliable and accurate and to reduce reporting times [22–26] and it is worth pointing out the encouraging results obtained for bone age estimation of the index finger alone (as opposed to the entire left hand and wrist radiograph) when using a neural-network-based AI application, which paves the way for hand-held bone age estimation machines [26].

While some authors have focused their work on AI determination of bone age from other sites, such as the pelvis [27] or knee [28], and other modalities, such as MRI [29–32], a relatively recent systematic review highlighted the lack of such studies, in addition to the need for more research assessing potential socioeconomic and ethnic variations on the performance of such AI tools [33].

## Emerging/future applications

### Bone health index

There is no reliable method of predicting fracture risk in children. While dual-energy X-ray absorptiometry is the gold standard for bone mineral density assessment in children, it has limitations [34–36]. As such, other quantitative bone imaging techniques have been developed including AI applications (predominantly related to adults; Table 1). Of relevance to pediatrics is radiogrammetry. Originally performed manually [37], this technique lends itself to automation because it measures cortical thickness of the phalanges in relation to their lengths, thereby producing an index of bone strength.

In addition to determining bone age, the BoneXpert software discussed in the previous section also performs “digital X-ray radiogrammetry,” providing an indication of bone health called the “bone health index” or “BHI” (Fig. 2 and Table 2). Bone health index is derived from a measurement of the cortical thickness, width and length of the three middle metacarpals. A standard deviation score is also provided, allowing comparison with the bone health index of healthy Caucasian children of the same age and gender.

While a few studies have been performed with favorable results [38–41], the clinical role of the bone health index in monitoring and assessing bone strength in children (of any ethnicity) has not been elucidated. In a recent systematic review, peripheral quantitative computed tomography (pQCT), bone health index and quantitative ultrasound (QUS) were compared with dual-energy X-ray absorptiometry. Meta-analysis showed BHI to have the strongest correlation with dual-energy X-ray absorptiometry, with a pooled estimate of correlation of 0.71 compared to 0.57 for both pQCT and QUS [42]. These results encourage further research into the potential clinical application of BHI.

### Fracture assessment

#### Appendicular fractures

The few studies that have assessed the utility of AI for appendicular fracture detection in children have predominantly concentrated on the elbow joint, possibly because of the complexity of the elbow joint and multiple unossified epiphyseal centers that are found in children. England et al. [43] used a relatively small set of lateral radiographs to train (657 images), validate (115 images) and test (129 images) a convolutional neural network for the identification of elbow joint effusions. Compared to the reference standard of radiologists’ reports, the network had sensitivity, specificity and accuracy of 0.91.

In a significantly larger study consisting of 21,456 anteroposterior and lateral elbow radiographs, Rayan et al. [44] determined the feasibility of deep learning to correctly classify elbow radiographs as normal or abnormal. The true positive rate (i.e., those radiographs correctly classified as abnormal) was highest for supracondylar fractures (0.996) and lowest for osteochondral lesions (0.000), although it should be noted that there were only two cases of osteochondral lesions in the entire dataset. Most recently, Choi et al. [45] assessed the ability of a convolutional neural network to correctly identify supracondylar fractures from 1,266 anteroposterior and lateral elbow radiographs.

The results of these three studies (summarized in Table 3; [43–45]) are encouraging. However, particularly given their relatively low positive predictive values, the current role of such AI tools would appear to be in the initial triage of elbow radiographs following trauma in children, in areas where a (pediatric) radiologist is not immediately available. Future research could also be directed toward determining the feasibility of AI tools for fracture detection in children at other appendicular sites, in a similar way to ongoing investigations in, e.g., the wrist [46] and proximal femur [47] of adults.

**Table 3:** Diagnostic accuracy of artificial intelligence (AI) applications for elbow trauma in children

Author [reference]	Sensitivity	Specificity	Accuracy	Positive predictive value	Negative predictive value
England et al. 2018 [43]	0.91	0.91	0.91	–	–
Rayan et al. 2019 [44]	0.91	0.84	0.88	0.87	0.89
Choi et al. 2020 [45] (temporal test set)	0.93	0.92	–	0.80	0.98
Choi et al. 2020 [45] (geographic test set)	0.10	0.86	–	0.70	0.10

#### Axial fractures

A decision tree might be seen as a flowchart-like structure, with each branch representing a potential outcome. Optimal trees are predictive AI algorithms that limit the number of outcomes while encompassing as much of the available data as possible [48]. Bertsimas et al. [49] used an optimal trees artificial intelligence approach to predict cervical spine trauma in children. However, this model was based on history and clinical parameters (including Glasgow Coma Scale) and used imaging interpretation by radiologists as an outcome measure for presence or absence of fracture, rather than using the algorithm to classify radiographs. Given the difficulty associated with obtaining adequate views (particularly in younger children) and complexity of the cervical spine [50], this would be a worthy field for the development of an AI diagnostic tool.

Other studies assessing AI tools for detecting vertebral fractures in children relate to osteoporotic compression fractures rather than post-traumatic fractures and are briefly reviewed next.

**Fig. 3:**
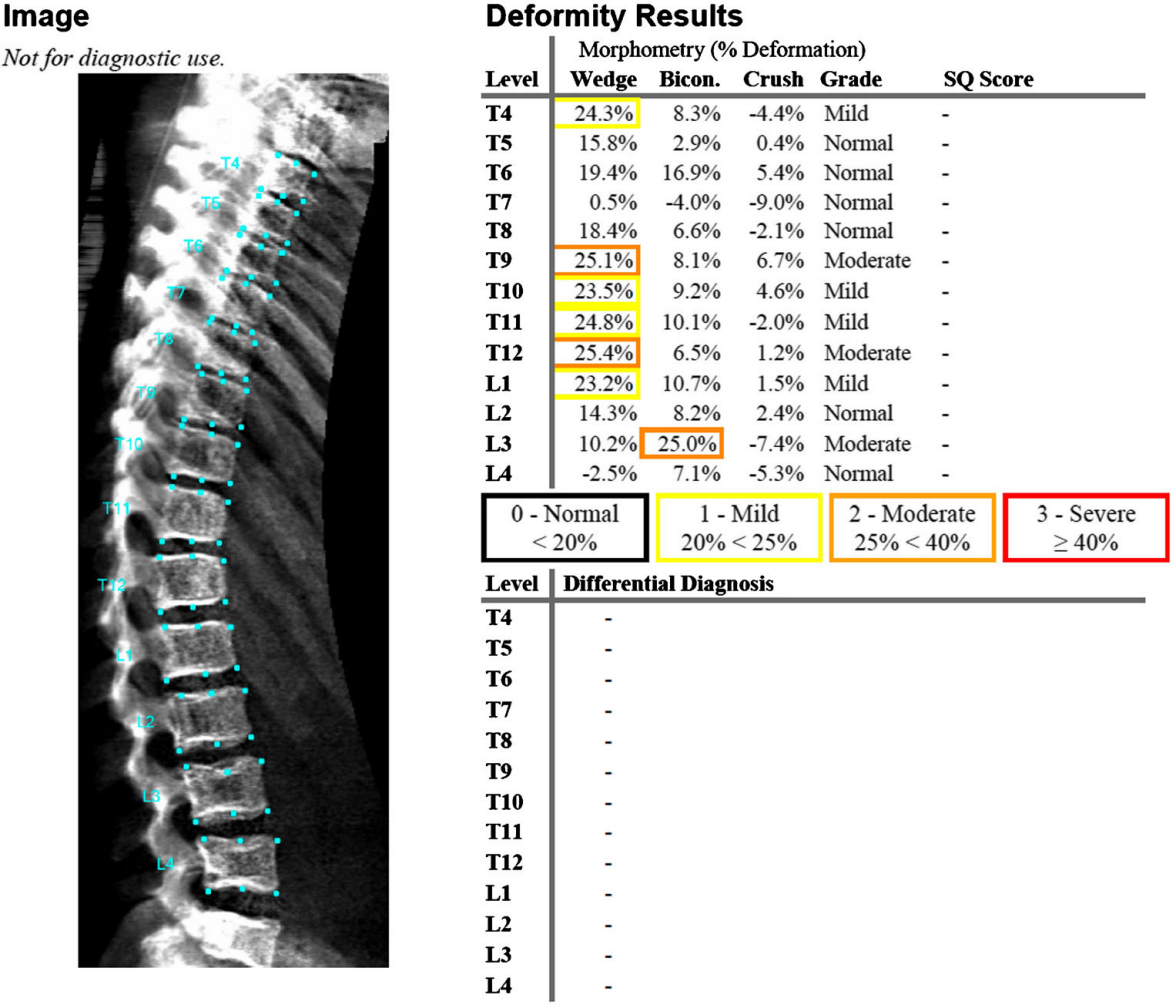
Lateral spine dual-energy X-ray absorptiometry scan in a 12-year-old boy, left, with deformity results right. Morphometric vertebral fracture assessment using SpineAnalyzer identifies four mild (T4, T10, T11, L1) and three moderate (T9, T12, L3) fractures. *Bicon.* biconcave, *SQ* semi-quantitative score (of Genant et al. [56])

The diagnosis of vertebral crush fractures from dual-energy X-ray absorptiometry scans is termed vertebral fracture assessment, and the Lunar iDXA machine (GE Healthcare Lunar, Buckinghamshire, UK) has been shown to be as reliable as radiographs for vertebral crush fracture diagnosis in children at a lower radiation dose penalty [51, 52]. The use of software tools to diagnose vertebral fractures from dual-energy X-ray morphometry scans is termed morphometric vertebral analysis [52]. Such software tools are widely available for clinical use in adults; however, they have not been licensed for use in children. Given both the wide variability in diagnosis of vertebral fractures in children [53] and that the recognition of vertebral shape (morphometry) lends itself to AI applications, researchers have assessed the accuracy and reliability of existing adult software, specifically SpineAnalyzer (Optasia Medical, Cheadle, UK) and AVERT (Optasia Medical) in the diagnosis of vertebral fractures in children [54–56]. The AI tools SpineAnalyzer and AVERT are semi-automated; they require an individual to identify and label the centers of vertebral bodies T4 to L4 (any non- or poorly visible vertebrae can be omitted). The tools then automatically outline the vertebral bodies using 6 (SpineAnalyzer) or 33 (AVERT) points and provide an output indicating normal or fractured vertebrae and severity of fracture based on height loss ratios (Fig. 3; [56]). The reader can manually reposition any points that were erroneously identified by the software. The conclusion of these studies is that the diagnostic accuracy of existing adult (semi-automated) software tools for vertebral fracture assessment in children is insufficiently adequate for clinical use (Table 4). Reasons for this include unossified ring apophyses, variation in vertebral shape with age and normal variants in children, all issues that are less problematic (if at all) in adults. The adult tools were trained using the radiographs of post-menopausal women and (despite the misleading final column in Fig. 3, which suggests otherwise) are based on the Genant et al. [56] classification for vertebral fractures. If morphometric vertebral analysis is to be accurate in children, then any tool developed must be trained using the spine radiographs of a cohort of healthy children [57].

**Table 4:** Diagnostic accuracy of artificial intelligence (AI) applications for morphometric vertebral fracture assessment in children^a^

Author [reference]	Sensitivity	Specificity	False-positive rate	False-negative rate	Kappa
Crabtree et al. 2017 [52] (6-point technique^b^)	0.79	0.71	0.13	0.27	0.24–0.60
Alqahtani 2019 [54] (SpineAnalyzer)	0.31	0.96	0.04	0.69	0.16–0.44
Alqahtani 2019 [54] (AVERT)	0.41	0.91	0.09	0.59	0.26–0.46
Alqahtani 2020 [55] (AVERT)					
Single radiographer Additional observers	0.80 0.89	0.90 0.79	0.10 0.21	0.20 0.11	- 0.29–0.69

^a^From dual-energy X-ray absorptiometry scans

^b^The precise software tool used was not specified

#### Inflicted fractures (child abuse)

Inflicted metaphyseal and rib fractures in infants and young children are often difficult to detect and yet are highly predictive of abuse [58, 59]. United Kingdom national guidelines (adopted by the European Society of Paediatric Radiology) advise that images be double-reported by at least one pediatric radiologist [60, 61]; therefore in centers where this is not possible because of staffing issues, it would be helpful to have an AI-assist tool, if only to highlight suspicious areas for closer review by the radiologist on either skeletal surveys performed for suspected abuse or (perhaps more important) on radiographs performed for other indications, e.g., a chest radiograph for cough. Work has been done on the AI-assisted detection of rib fractures in adults, with encouraging results [62–65], including the development of fully automated convolutional neural networks to perform this task [66, 67]. However, to the author’s knowledge, no such studies have been carried out for suspected abusive fractures in infants and young children. Research in this area should be encouraged.

To assist radiologists and others in the field, a web-based tool to unify the investigative protocol in suspected abuse and to support training and multicenter national and international research, a knowledge base to be populated with clinical information, radiographs and radiographic information has been described [68] and continues to be developed (ongoing work of author).

### Other emerging/future pediatric musculoskeletal applications

#### Developmental hip dysplasia

Two studies have assessed the ability of neural networks to diagnose developmental hip dysplasia in children [69, 70]. Li et al. [69] used a training set of 11,473 anteroposterior pelvic radiographs and a test set of 101 images for the diagnosis of developmental dysplasia of the hip based on the Sharp angle (acetabular index). They found that accuracy was similar when compared to orthopedic surgeons and required less time, and they concluded that their AI tool could potentially replace orthopedic surgeons [69]. Zhang et al. [70] used 9,081 anteroposterior pelvic radiographs as their training set and a further 1,138 anteroposterior pelvic radiographs as their test set for the diagnosis of developmental hip dysplasia based on the acetabular index. They concluded that their deep-learning system improved consistency, convenience and effectiveness compared to clinician-led diagnosis and suggested that it might simplify current screening pathways [70].

As far as can be ascertained, neither of these tools was compared to a reference standard of pediatric radiologists. In the author’s opinion, this would be an important next step before widespread use of such AI tools by pediatric radiology departments.

#### Spinal alignment

Several studies have been conducted to determine the degree of scoliosis and other measurements of the spine using conventional radiographic images [71], biplanar radiographic images [72] or moiré images [73]. All such studies have shown promising results and indeed automated spine and lower limb measurements are performed by the biplanar imaging system installed at the author’s institution.

Other authors have assessed the ability of AI to predict scoliosis progression [74], assess the Risser stage [75], detect evidence of scoliosis treatment from radiographs [76] and to automate three-dimensional (3-D) spine reconstructions from biplanar images [77].

#### Miscellaneous

A few other studies conducted in children (or children and adults) and assessing AI applications in the musculoskeletal system are worthy of mention and include determining leg-length discrepancy from radiographs [78], quantifying the degree of metopic craniosynostosis from skull CT scans [79], predicting the presence of discoid lateral menisci from radiographs [80], determining muscle mass from dual-energy X-ray absorptiometry scans in cerebral palsy [81] and discerning sexual dimorphism from hand and wrist radiographs [82]. Further applications of some of these tools are obvious, e.g., diagnosis of premature fusion of sutures other than the metopic suture and determination of muscle mass in other conditions such as myopathies and juvenile dermatomyositis. The clinical utility of a tool that identifies gender from hand and wrist radiographs is limited to forensic imaging, perhaps helping with the identification of bodies destroyed by mass disasters, but it is significant because (to the author’s knowledge) it is the only example of a potential AI-extend tool in pediatric musculoskeletal imaging (i.e. a tool that performs a task over and above the capability of radiologists).

Computer-assisted diagnosis of skeletal dysplasias might be based both on AI-assisted morphological analysis and on the creation of “ontologies” in the skeletal dysplasia domain. An ontology organizes large datasets into sets of categories/concepts and forms relationships between them [83]. Ontologies related to skeletal dysplasias include the Human Phenotype Ontology [84], the Bone Dysplasia Ontology [85] and the dynamic Radiological Electronic Atlas of Malformation Syndromes (dREAMS) [86].

Pertaining to AI-assisted diagnosis of skeletal dysplasias based on skeletal morphometry, preliminary work using radiographs of infants from the dREAMS database has shown an accuracy of 78.0% to 87.5% for lateral spine, 68.0% to 75.0% for anteroposterior spine and 87.5% to 88.0% for anteroposterior chest radiographs in dichotomizing images to “achondroplasia” or “not achondroplasia” categories [87]. Accuracy and confidence intervals would be expected to improve using a dataset larger than that used by the authors (40 lateral spine, 16 anteroposterior spine and 26 anteroposterior chest radiographs in a ratio of 70% to 30% for training and testing, respectively). Nevertheless, the results provide proof of concept and suggest that the task is worth pursuing.

## Conclusion

Bone age assessment tools are the only pediatric musculoskeletal AI tools available on the market. In recent years, increasing research has been conducted in areas such as elbow fractures, developmental hip dysplasia and scoliosis assessment. However, there is significant scope for more work, particularly in areas such as the diagnosis of vertebral fractures, inflicted injury, skeletal dysplasias and musculoskeletal oncology. Pediatric radiologists are encouraged to be members of the research teams conducting such studies, so that the reference standard used is the diagnostic accuracy of pediatric radiologists, rather than the diagnostic accuracy of clinicians from other specialties, which is the case in some publications. AI tools will not replace the pediatric musculoskeletal radiologist in the near future, if ever.
